# Alexithymic traits can explain the association between puberty and symptoms of depression and anxiety in adolescent females

**DOI:** 10.1371/journal.pone.0210519

**Published:** 2019-01-16

**Authors:** Renske van der Cruijsen, Jennifer Murphy, Geoffrey Bird

**Affiliations:** 1 Department of Developmental Psychology, Leiden University, the Netherlands, Leiden, The Netherlands; 2 MRC Social, Genetic and Developmental Psychiatry Centre, Institute of Psychiatry, Psychology and Neuroscience, King’s College London, London, United Kingdom; 3 Department of Experimental Psychology, University of Oxford, Oxford, United Kingdom; Universita Cattolica del Sacro Cuore Sede di Roma, ITALY

## Abstract

Symptoms of internalizing disorders such as depression and anxiety increase in adolescence, especially in females. However, gender differences in depression and anxiety symptoms emerge only after puberty onset. Levels of alexithymia, characterized by difficulties identifying and describing one’s emotions, are elevated in depression and anxiety, and fluctuate across adolescence in a gender-specific manner. This study investigated changes in alexithymia across adolescence, and explored the potential role of alexithymia in the development of depression and anxiety, separately for females and males. Accordingly, 140 adolescents aged 11 to 21 years (77 female) completed self-report measures of alexithymia, depression and anxiety, and pubertal development. For females alone, pubertal maturation was associated with alexithymic traits (specifically difficulties identifying and describing feelings), as well as symptoms of depression and anxiety. After accounting for alexithymia, the relationship between puberty and depression and anxiety was absent or reduced in females. Thus, alexithymic traits may have differential consequences for males and females, and possibly contribute towards increased depression and anxiety symptoms in females during adolescence. We propose that developmental changes in alexithymia should be considered when studying the onset and development of internalizing psychological disorders during adolescence.

## Introduction

Adolescence is a developmental period associated with considerable physical, social, and psychological change [1–3]. It is also the period within which a number of psychiatric conditions, including depression and anxiety, are most likely to have their onset [4,5]. These observations have prompted the suggestion that the changes experienced in adolescence (for example changes related to puberty) may confer vulnerability for the development of psychiatric conditions, rather than, or in addition to, chronological age [6,7].

It has previously been suggested that the onset of psychopathology, and increased levels of subclinical depression and anxiety, may be explained by age, pubertal status (i.e. stage of maturation), and pubertal timing (i.e. stage of maturation relative to same-aged peers [8]). With respect to age, studies demonstrate age-dependent increases in depressive symptoms across adolescence [9–11], whereas symptoms of anxiety have been found to decrease in early adolescence (age 10 to 13–14), and rise again from age 14–15 [12,13]. With respect to puberty, multiple studies indicate that both pubertal status and pubertal timing are related to symptoms of depression and anxiety [6,14,15], and to the onset of clinical depression and anxiety disorders [7,16–18]. These results highlight the necessity of accounting for pubertal stage and pubertal timing in addition to age when investigating the impact of adolescence on psychiatric symptoms.

Effects of puberty appear to interact with gender; although females experience depression and anxiety more often than males, these gender differences only emerge after the onset of puberty [3,19–22]. Indeed, when directly compared, it was found that pubertal status predicted the emergence of depression-related gender differences better than age [20]. Moreover, early pubertal timing has been associated with symptoms of depression and anxiety in females [21,23,24], whereas in males, late pubertal timing has been related to disruptive behaviour and symptoms of depression [21,23,25].

It can be seen therefore, that all three developmental measures (age, pubertal stage, and pubertal timing) have previously been related to an increase in symptoms of depression and anxiety in adolescence, with specific effects of puberty being dependent upon gender. Although evidence for a link between adolescent development and anxiety and depression is substantial, at present the mechanism by which age and pubertal factors increase vulnerability to depression and anxiety is unknown. This study aims to ascertain whether alexithymia has the potential to explain the link between adolescent development and depression and anxiety.

### Alexithymia, psychopathology, and adolescence

Alexithymia (or: pensée operatoire, as it is often described in French-language literature [26] is a sub-clinical condition characterised by an inability to identify and describe emotions, together with an externally oriented thinking style [27]. Alexithymic traits are therefore typically measured across three subscales: difficulty identifying feelings (DIF), difficulty describing feelings (DDF), and externally oriented thinking (EOT). The clinical relevance of alexithymia is increasingly being recognised [28], not least because increased rates of alexithymia are observed in both adolescents and adults with a range of psychiatric disorders; for example eating disorders [29,30], schizophrenia [31,32], and Autism Spectrum Disorder (ASD) [33,34]. Of relevance to the current study is that alexithymia, specifically the subscales relating to difficulties identifying and describing feelings, is often associated with symptoms of depression and anxiety [35–39].

Also of relevance is the impact of adolescence on rates of alexithymia, and how this is moderated by gender. The overall prevalence of alexithymia is higher in adolescents (18%) [40] than in adults (8–10%) [41]; with higher rates in early adolescence, decreasing to adult levels by late adolescence [40,42]. Although several studies show higher rates of alexithymia in males compared to females in adult samples [37,43], this pattern is reversed in adolescence [36,44]. During adolescence, levels of alexithymia are age- and gender-dependent: total alexithymia scores and DDF scores show a negative relationship with age over adolescence in males, whereas total alexithymia, DIF, and DDF scores remain more stable in females [40]. To our knowledge, however, no studies have investigated the development of alexithymia scores in relation to pubertal status or pubertal timing.

The link between adolescence and alexithymia, and between alexithymia and depression and anxiety, makes it possible that alexithymic traits can explain the link between adolescent development and depression and anxiety. Based on existing research, it is unclear whether any effect will be between age, puberty stage or pubertal timing and depression/ anxiety, although there is reason to believe that these relationships (especially those involving pubertal factors) may be gender-specific.

### Current study

The present study aimed to 1) investigate the effect of age, pubertal status, and pubertal timing on alexithymia, 2) identify the relationship between age, pubertal status, pubertal timing, and depression and anxiety, and 3) examine whether alexithymia has the potential to explain the relationship between age/pubertal status/pubertal timing, and symptoms of depression and anxiety.

These relationships were separately examined for each of the three alexithymia subscales as evidence suggests that they are differentially related to clinical outcomes. Although there are high correlations between DIF and DDF scores, correlations between these subscales and EOT scores are lower [45]. Additionally, DIF and DDF scores but not EOT scores are related to depression and anxiety [35,38,39]. Analyses were conducted separately for males and females due to the evidence reviewed above that pubertal influences on depression and anxiety vary according to gender (e.g. [19,22]), the greater incidence of depression and anxiety in adolescent females in comparison to males (e.g. [21,22]), and the fact that pubertal status reflects different bodily changes in males and females.

In line with previous evidence we predicted that symptoms of depression and anxiety would increase with age (e.g. [10,12]) and pubertal status [6,14,15], and that early pubertal timing would relate to higher depression and anxiety scores than late pubertal timing in females [21, 24, 46–48]. Additionally, we predicted that any shared variance between alexithymia and age/pubertal status/pubertal timing when predicting symptoms of anxiety or depression would be specific to the DIF and DDF subscales, since these have previously been found to relate to depression and anxiety [35,36,39].

## Methods

### Participants

A total of 160 adolescents participated in this study, of whom 20 were excluded due to incomplete puberty assessments. Accordingly, a total of 140 healthy adolescents (77 female) were included in analyses. Ages ranged between 11.0 and 20.9 years in females (*M*_*age*_ = 15.97, *SD* = 3.0), and 11.0 to 20.7 years in males (*M*_*ag*e_ = 16.21, *SD* = 2.9). Pubertal stage was measured on a continuous scale with possible scores from 5 to 20, indicating individuals for whom puberty has not yet begun, and individuals for whom pubertal development is complete, respectively. In females, pubertal status scores ranged from 5 to 20 (mean Pubertal status score (PS) = 15.3, *SD* = 3.5), and from 5 to 20 in males (mean PS = 14.3, *SD* = 4.2) (see Table 1 for more details).

**Table 1:** Number of Participants per age, PS score and gender as included in the final sample.

Age	Mean PS score			Number of participants		
	Females	Males	Total	Females	Males	Total
**11years**	8.6	7.6	8.3	10	5	15
**12 years**	12.2	10.3	11.3	6	6	12
**13 years**	16.4	9.6	13.0	7	7	14
**14 years**	15.0	15.4	15.2	7	7	14
**15 years**	17.0	13.8	15.8	9	5	14
**16years**	16.0	14.8	15.5	7	6	13
**17years**	17.0	18.3	17.5	9	6	15
**18years**	16.1	17.6	16.9	7	7	14
**19years**	18.1	16.1	17.1	7	7	14
**20years**	17.6	17.7	17.7	8	7	15

*Note*. PS = Pubertal Status

Participants were recruited via talks at schools: adolescents could indicate their interest in participating in scientific research via www.juniorhersenen.nl. Prior the start of this study, we selected adolescents within the intended age-range and called them (and parents of minors) to inform them about the study and ask whether they would be interested in participating. All adolescents who decided to participate, and parents of minors, gave written informed consent before inclusion in the study. No participants were diagnosed with depression or anxiety at the time of the study. The study was approved by the Medical Ethics Committee (CME) of the Leiden University Medical Centre (LUMC). All procedures performed in studies involving human participants were in accordance with the ethical standards of the institutional and/or national research committee and with the 1964 Helsinki declaration and its later amendments or comparable ethical standards.

### Measures

Descriptive information for each measure, and statistical differences between females and males on these measures, can be found in Table 2.

**Table 2:** Descriptive statistics of all measures, and statistical differences between females and males on these measures.

	Females		Males		
	*M*	*SD*	*M*	*SD*	*U*
Age	15.97	3.00	16.21	2.89	2320.0
Pubertal Status^a^	15.30	3.54	14.33	4.22	
Pubertal Timing^a^	-.05	2.58	-.02	2.89	
Alexithymia	34.47	5.32	33.22	5.95	
DIF	11.70	2.98	10.19	2.83	1645.5**
DDF	9.51	2.47	8.99	2.79	2106.5
EOT	13.26	2.88	14.05	2.77	1992.0
RCADS	28.05	15.28	20.10	10.64	
MD	5.88	3.71	5.22	3.86	2116.0
GA	5.23	2.64	3.83	2.14	1647.5**
SP	8.87	4.90	5.89	3.61	1491.5***
PD	3.30	3.03	1.75	1.82	1668.0**
SA	2.69	2.23	1.37	1.51	1464.0***
OCD	2.08	2.39	2.05	1.96	2336.0

*Note*. Pubertal status = scores on the pubertal development scale; higher scores indicate a more mature pubertal state. Pubertal timing = pubertal development relative to same-aged peers; positive scores indicate a relatively early pubertal timing, negative scores indicate a relatively late pubertal timing, Alexithymia subscales: DIF = Difficulty Identifying Feelings, DDF = Difficulty Describing Feelings, EOT = Externally Oriented Thinking. RCADS = Revised Child Anxiety and Depression Scale, subscales: MD = Major Depression, GA = Generalized Anxiety, SP = Social Phobia, PD = Panic Disorder, SA = Separation Anxiety, OCD = Obsessive Compulsive Disorder. To test for significant differences between females and males on all measures a Mann-Whitney U-test was performed. *U* = the value of Mann-Whitney’s U test.

*** Difference between females and males is significant at p < .001,

** p < .01,

* p < .05 (2-tailed).

^a^Note that it is not appropriate to compare pubertal measures in males and females as scores index different processes and are scored differently.

#### Pubertal status

The Pubertal Development Scale (PDS; [49]) was used to measure pubertal status. The internal reliability of this scale in this sample was assessed using Cronbach’s alpha (females: ɑ = .72, males: ɑ = .86). This scale assesses changes in body hair, skin changes, and growth rate in females and males. Gender-specific pubertal changes are also quantified; voice changes and facial hair growth in males, and breast growth and menarcheal status in females. The items are rated on a scale of 1 to 4 (1 = no development, 2 = development has just begun, 3 = development is definitely underway, and 4 = development is complete). The menarche item is rated 1 (no) or 4 (yes). The total PDS score was used in analyses.

#### Pubertal timing

To calculate pubertal timing, we regressed PDS scores onto age in this sample. The regression equation was used to estimate the expected PDS score based on age (for females: *r* = .68, *p* < .001; Y = 0.82x + 2.56; for males: *r* = .73, *p* < .001; Y = 1.07x−2.38). Subsequently, we calculated pubertal timing for each participant by subtracting the expected PDS score (based on age) from the actual PDS score [50–52]. As a result, positive scores on pubertal timing indicate a relatively early pubertal timing, whereas negative scores indicate a relatively late pubertal timing for the participant’s age.

#### Alexithymia

To measure alexithymia, participants completed the Alexithymia Questionnaire for Children (AQC; [53]). Like the adult version [54], this scale consists of 20 items assessing the three alexithymia subscales: Difficulty Identifying Feelings (DIF; e.g. ‘I am often confused about what emotion I am feeling’), Difficulty Describing Feelings (DDF; e.g. ‘It is difficult for me to find the right words for my feelings’), and Externally-Oriented Thinking (EOT; e.g. ‘I prefer to just let things happen rather than to understand why they turned out that way’). The items are rated on a scale of 1 to 3 (1 = not true, 2 = a bit true, 3 = true). The internal reliability of the subscales in this sample was assessed using Cronbach’s alpha (DIF: ɑ = .79, DDF: ɑ = .76, EOT: ɑ = .59).

#### Depression / Anxiety

To assess symptoms of depression and anxiety participants completed the Revised Child Anxiety and Depression Scale (RCADS; [55]). This scale consists of 47 items and is specifically developed to measure DSM-IV symptoms of major depression (MD), generalized anxiety (GA), social phobia (SP), panic disorder (PD), separation anxiety (SA), and obsessive-compulsive disorder (OCD) in adolescents. The items are rated on a scale of 0 (never) to 3 (always). The internal reliability of the subscales was assessed using Cronbach’s alpha (MD: ɑ = .80, GAD: ɑ = .68, SP: ɑ = .85, PD: ɑ = .73, SA: ɑ = .63, OCD: ɑ = .67). Participants’ minimum, maximum and mean scores on these measures, plus the lowest and highest possible scores, can be found in S1 Table.

### Procedure

This study was part of a larger study (the Leiden Self-Concept study) that consisted of two parts. First, participants completed the RCADS at home. Second, participants visited the lab where they completed the PDS, and the AQC. Questionnaires were completed no longer than one week before the lab visit.

### Statistical analyses

All analyses were performed separately for females and males. First, we examined the intercorrelations between the developmental factors (age, pubertal status, and pubertal timing) and alexithymia, depression, and anxiety. Second, where a relationship between developmental factors and depression/anxiety symptoms was found, we also tested for effects of the three alexithymia subscales on the relationship between the developmental factor and depression and anxiety using hierarchical regression analyses to see whether alexithymic traits have the potential to explain some of the relationships between developmental factors and psychiatric symptoms. Because of the relatively skewed distributions of the residuals, especially within the analyses performed in males (see plots in S1 Fig), we performed subsequent entry method robust regressions to confirm our results [56]. Robust regressions were conducted in Matlab 2014a and the default tuning function (‘bisquare’; 4.685) was utilised. This regression method down weights the influence of extreme data points, reducing the influence of data points that can exert a large influence on the results derived from the least-squares method. When data is normal, the robust and least-squares methods produce very similar results (see [56]).

## Results

Where parametric tests are used, all required assumptions were met. To test for possible differences in alexithymia scores between females and males, a Mann-Whitney U-test was performed (Table 2). Results showed that females (*M* = 11.7, *SD* = 3.0) indicate more difficulties identifying feelings than males (*M* = 10.2, *SD* = 2.8; *U* = 1645.5, *p* = .001). Alexithymia ratings on the other two subscales did not differ between males and females (all *p*-values >.07).

### Development of alexithymia symptoms

Table 3 reports the correlations between all alexithymia subscales and age, pubertal status, and pubertal timing, separately for females and males. In females, EOT correlated negatively with age and pubertal status, whereas DIF and DDF correlated positively with pubertal status and pubertal timing. Furthermore, total alexithymia score correlated positively with pubertal timing. In males, EOT correlated negatively with age, pubertal status, and pubertal timing.

**Table 3:** Correlations between age, pubertal status and pubertal timing, and alexithymia subscales.

	Females			Males		
	Age	Pubertal Status	Pubertal Timing	Age	Pubertal Status	Pubertal Timing
**DIF**	.075	.257*	.282*	.114	.224	.207
**DDF**	.115	.315**	.324**	.136	.214	.168
**EOT**	-.462**	-.299**	.026	-.369**	-.461**	-.280**

*Note*. Pubertal status = scores on the pubertal development scale; higher scores indicate a more mature pubertal state. Pubertal timing = pubertal development relative to same-aged peers; positive scores indicate a relatively early pubertal timing, negative scores indicate a relatively late pubertal timing, Alexithymia subscales: DIF = Difficulty Identifying Feelings, DDF = Difficulty Describing Feelings, EOT = Externally Oriented Thinking.

**Correlation is significant at p < .01 (2-tailed).

*Correlation is significant at p < .05 (2-tailed).

### Development of symptoms of depression and anxiety

Table 4 describes the relationship between the RCADS subscales and age, pubertal status, and pubertal timing. In females, symptoms of depression correlated positively with pubertal status and pubertal timing. Symptoms of social phobia correlated positively with pubertal status, while symptoms of generalized anxiety disorder correlated positively with pubertal status and age. In males, symptoms of generalized anxiety disorder were positively correlated with age as in females. Separation anxiety correlated negatively with pubertal status and pubertal timing.

**Table 4:** Correlations between age, pubertal status and pubertal timing, and symptoms of depression and anxiety.

	Females			Males		
	Age	Pubertal Status	Pubertal Timing	Age	Pubertal Status	Pubertal Timing
**MD**	.164	.330**	.299*	.002	-.002	-.005
**GA**	.268*	.319**	.185	.254*	.088	-.142
**SP**	.131	.229*	.191	.171	.032	-.136
**PD**	.063	.145	.140	-.137	-.188	-.129
**SA**	-.133	.032	.170	-.236	-.420**	-.363**
**OCD**	-.031	.069	.123	-.082	-.076	-.024

*Note*. Pubertal status = scores on the pubertal development scale; higher scores indicate a more mature pubertal state. Pubertal timing = pubertal development relative to same-aged peers; positive scores indicate a relatively early pubertal timing, negative scores indicate a relatively late pubertal timing, MD = Major Depression, GA = Generalized Anxiety, SP = Social Phobia, PD = Panic Disorder, SA = Separation Anxiety, OCD = Obsessive Compulsive Disorder

**Correlation is significant at p < .01 (2-tailed).

*Correlation is significant at p < .05 (2-tailed).

### Relationship between alexithymia and symptoms of depression and anxiety

Table 5 presents correlations between the alexithymia and RCADS subscales. In females, DIF correlated positively with all RCADS subscales. DDF correlated positively with RCADS subscales major depression, generalized anxiety, and social phobia, and EOT correlated negatively with generalized anxiety. In males, DIF correlated positively with all RCADS subscales except separation anxiety. DDF correlated positively with RCADS subscales major depression, generalized anxiety, social phobia, and panic disorder, and EOT correlated positively with separation anxiety in males.

**Table 5:** Correlations between alexithymia subscales, and depressive and anxiety symptoms.

	Females			Males		
	DIF	DDF	EOT	DIF	DDF	EOT
**MD**	.529**	.227*	-.152	.488**	.392**	.085
**GA**	.542**	.270*	-.230*	.407**	.280*	.029
**SP**	.490**	.352**	-.173	.269*	.319*	.036
**PD**	.385**	.157	-.128	.354**	.266*	.117
**SA**	.331**	.079	.148	.116	.128	.282*
**OCD**	.510**	.125	.031	.316*	.175	.080

*Note*. Subscales of alexithymia: DIF = Difficulty Identifying Feelings, DDF = Difficulty Describing Feelings, EOT = Externally Oriented Thinking. Anxiety/Depression ratings: MD = Major Depression, GA = Generalized Anxiety, SP = Social Phobia, PD = Panic Disorder, SA = Separation Anxiety, OCD = Obsessive Compulsive Disorder.

**Correlation is significant at p < .01(2-tailed).

*Correlation is significant at p < .05 (2-tailed).

### Effects of alexithymia in a hierarchical regression model

Hierarchical regression analyses were used to test whether alexithymic traits have the potential to contribute towards, or explain, the relationship between developmental factors (age/pubertal status/pubertal timing) and symptoms of depression and anxiety. Analyses were only performed where there were significant correlations between alexithymia and developmental factors, alexithymia and symptom levels, and developmental factors and symptom levels. Note that separate analyses were performed for each developmental variable (age, pubertal status, pubertal timing), and for each of the different RCADS subscales, where the pattern of zero-order correlations made an effect of alexithymia possible.

#### Females

In females, we tested the effect of adding alexithymia to regression models testing the relationship between age and generalized anxiety symptoms. Next, we tested the effect of adding alexithymia to regression models testing the relationship between pubertal status and major depression, generalized anxiety symptoms, and social phobia scores. Subsequently, we tested the effect of adding alexithymia to models testing the relationship between pubertal timing and depression (see S2 Table).

Adding alexithymia subscales in the second step of the regression improved all tested models: the regression of generalized anxiety on age (*R*^2^change = .29, *F*change = 10.70, *p* < .001), the regression of major depression symptoms (*R*^2^change = .23, *F*change = 8.18, *p* < .001), generalized anxiety symptoms (*R*^2^change = .25, *F*change = 9.38, *p* < .001), and social phobia symptoms (*R*^2^change = .22, *F*change = 7.23, *p* < .001) on pubertal status, and the regression of major depression symptoms on pubertal timing (*R*^2^change = .24, *F*change = 8.69, *p* < .001).

In these five models, only DIF was a significant predictor of depression and anxiety symptoms (age—generalized anxiety: *t* = 4.94, *p* < .001; pubertal status—major depression: *t* = 4.74, *p* < .001; pubertal status—generalized anxiety: *t* = 4.74, *p* < .001; pubertal status—social phobia: *t* = 3.48, *p* = .001; pubertal timing—major depression: *t* = 4.67, *p* < .001). Only in the regression model predicting major depression symptoms from pubertal status did the developmental factor remain a significant predictor in the model when alexithymia scales were included (pubertal status: *t* = 2.01, *p* = .048). Conversely, when alexithymia subscales were added in the first step, and the developmental factor was added in the second step, only in the model predicting major depression symptoms from pubertal status did the developmental factor significantly increase the amount of variance explained (*R*^2^ change = .04, *F* change = 4.04, *p* = .048; in the other models, all *p*-values > .06).

#### Males

In males we tested the effect of adding alexithymia to regression models testing the effect of 1) pubertal status, and 2) pubertal timing on separation anxiety (see S2 Table). Adding alexithymia subscales in the second step of the regression did not improve these models: the regression of pubertal status (*R*^2^ change = .066, *F* change = 1.68, *p* = .181) and pubertal timing (*R*^2^ change = .071, *F* change = 1.71, *p* = .175) on symptoms of separation anxiety. In these models, none of the alexithymia subscales were significant predictors of separation anxiety symptoms (pubertal status—separation anxiety: all *p*-values < .26; pubertal timing—separation anxiety: all *p*-values < .23). Conversely, when alexithymia subscales were added in the first step, and the developmental factor was added in the second step, in both models the developmental factor significantly increased the amount of variance explained (pubertal status: *R*^2^ change = .15, *F* change = 11.70, *p* = .001; pubertal timing: *R*^2^ change = .11, *F* change = 8.18, *p* = .006).

#### Robust regressions

To confirm the above regression analyses, we conducted additional robust analyses. As it is not possible to conduct hierarchical regressions in these analyses, we performed two separate analyses for each of the above tested models: one with only the developmental factor as a predictor, and one with the developmental factor and the alexithymia subscales as predictors. These analyses confirm the results described above.

In females, the developmental factor was significant in all five models when added as the only predictor (modelling generalized anxiety as a function of (1) age and (2) pubertal status, modelling major depression from (3) pubertal status and (4) pubertal timing, and modelling social phobia as a function of (5) pubertal status; all *p*-values < .018). In all five models, the developmental factor was not significant when the three alexithymia subscales were added as additional predictor variables (all *p*-values>.07; see S3 Table). In all models including both the developmental factor and alexithymia subscales, DIF contributed significantly to the outcome variable (all *p*-values < .002). Only in the model with social phobia did EOT additionally (negatively) predicted this outcome variable (*p* = .007). In males, the developmental factor was significant in both models when added as the only predictor (modelling separation anxiety as a function of (1) pubertal status and (2) pubertal timing; both *p*-values < .026), and these factors remained significant in the models including alexithymia predictor variables (both *p*-values < .013; see S4 Table). None of the alexithymia subscales significantly contributed to separation anxiety (all *p*-values>.18).

The use of mediation analyses with cross-sectional data for making casual inferences has been questioned [57,58]. However, as we aimed to identify a potential mechanism accounting for developmental influences on depression and anxiety, mediation analyses could be considered to be an appropriate alternative way to test our hypotheses. Therefore, we confirmed our previously described results using mediation analyses, and present these analyses in the Supporting Information (S1 File).

## Discussion

The main goals of this study were to explore the effect of developmental factors including age, pubertal development, and pubertal timing on levels of alexithymia, and to test whether alexithymia has the potential to explain the relationship between developmental factors and symptoms of depression and anxiety. As predicted, we found that these relationships were largely moderated by gender, although symptoms of depression and anxiety were related to DIF in both males and females. Only in females did alexithymia have the potential to explain the effect of development on depression and anxiety; the inclusion of DIF as a predictor in the regression analyses reduced the size (and statistical significance) of the association between pubertal status and pubertal timing and symptoms of depression, and the relationship between pubertal status and generalized anxiety and social phobia symptoms. The discussion is organized according to these findings. Each section first describes common results for females and males, before discussing results unique to females and then to males.

### Developmental effects on levels of alexithymia

Developmental effects on levels of alexithymia varied as a function of gender and alexithymia subscale. While externally-oriented thinking decreased with age and pubertal status in both females and males, increasing pubertal maturity was related to increasing difficulties in identifying and describing feelings in females. Earlier pubertal timing was related to increased difficulties identifying and describing feelings in females, whereas in males it was associated with less externally oriented thinking. Together, these results show that puberty is an important factor to consider when examining the development of alexithymia across adolescence [59], and add nuance to previous findings of a decrease in levels of alexithymia across adolescence [40,42].

### Developmental effects on symptoms of depression and anxiety

Symptoms of generalized anxiety, but not depression, increased with age in both males and females, and symptoms of depression, generalized anxiety, and social phobia increased with pubertal status in females. These results are partly in line with previous studies that found an increase in both anxiety and depression across adolescence in both males and females (e.g. [6,10,12,14,15]). Previous studies show greater increases in symptoms of depression in females compared to males, which is consistent with our finding that symptoms of depression significantly increase with pubertal development in females only.

As predicted, females who mature earlier showed more symptoms of depression compared to late maturing females [21,24]. However, in contrast to previous studies, in these data early maturing females did not show more anxiety symptoms compared to late maturing females [46,47]. Unlike females, in males, less mature pubertal status and late pubertal timing were related to increased symptoms of separation anxiety. This result complements previous studies indicating a decline of separation anxiety symptoms [60] in adolescence, and a drop in separation anxiety prevalence after childhood, with rates remaining stable in adolescence [61]. In combination with previous work, these data therefore suggest that pubertal status and pubertal timing confer a unique developmental trajectory unrelated to age with respect to separation anxiety across adolescence, at least in males.

### Relationship between alexithymia and symptoms of depression and anxiety

In line with previous studies that show higher alexithymia rates among adolescents with psychological disorders [36–39], we found that females and males who have more difficulty identifying feelings, also had more symptoms of depression and anxiety. The same relationship was found for DDF, but this subscale of alexithymia correlated with fewer anxiety subtypes than DIF. EOT was only related to one subtype of anxiety symptoms in females and males, which confirms previous studies indicating that it is predominantly difficulties identifying and describing feelings that are related to symptoms of depression and anxiety [36,39].

### Difficulty identifying feelings has the potential to explain the relationship between puberty and symptoms of depression and anxiety

We predicted that the potential of alexithymia to explain the impact of pubertal factors on depression and anxiety would be restricted to the DIF or DDF subscales, since these subscales have previously been found to relate to depression and anxiety. In line with our predictions, across five hierarchical regression analyses, results were consistent with the idea that DIF has the potential to explain the effect of puberty on levels of depression and anxiety. Additional mediation analyses supported this conclusion. However, as this is the first study investigating this possible mechanism, future studies should aim to replicate these results, preferably using a longitudinal design.

Specifically, we found that DIF was able to explain some of the effect of pubertal status (both DIF and pubertal status were significant predictors in the regression) and all of the effect of pubertal timing (only DIF remained a significant predictor in the regression model) on depression. Moreover, DIF explained the relationship between pubertal status and generalized anxiety, social phobia, and separation anxiety symptoms (pubertal status was not a predictor after the inclusion of DIF). Whilst causal mechanisms cannot be inferred from these data, results are consistent with a model whereby a more mature pubertal status / earlier pubertal timing results in an increase in DIF, which in turn results in more severe symptoms of depression and anxiety. Future longitudinal studies could aim to test these models. These effects were only present in females. Thus, results are consistent with the hypothesis that alexithymia plays an important role in the increased depression and anxiety seen in adolescent females. Though future longitudinal research is required to determine the casual relationship between DIF, pubertal status and symptoms of depression and anxiety, these data suggest such research is warranted.

The explanatory power of alexithymia, in particular of DIF, may at least in part help to explain the gender differences in levels of depression and anxiety during adolescence (e.g. [14,21,22]). While it was possible to account for the effect of developmental factors on symptoms of depression and anxiety via increases in DIF this effect was specific to females; DIF did not increase with age or pubertal status in males, and females gave higher DIF ratings than males. Although this hypothesis concerning the gender difference in the development of these psychological symptoms across adolescence is supported by the current data, it is clear that future studies should further investigate the precise role of alexithymia in the development of psychological symptoms in adolescence using a longitudinal design, taking into account other factors that may be involved with the onset of these symptoms. For example, one’s self-concept [2] and self-esteem [62] change considerably from childhood, across adolescence, and into adulthood, and these changes could also be related to an increase in symptoms of depression and anxiety [63–65].

### Limitations and future directions

Despite the potential of these findings to elucidate the relationship between adolescence and increased depression and anxiety, it is important to acknowledge certain limitations of the study. First, the mean pubertal status score was relatively high (15.3 for Females, 14.3 for males). Although pubertal development scores ranged from 5 to 20 (full range of scores) in both males and females, relatively few participants were in the early stages of puberty. Future research should include participants in an even broader age range, in order to make sure that the early pubertal stages are not underrepresented.

Second, pubertal timing was measured by comparing one’s pubertal status to that of same-aged peers within our sample of participants. Although other studies have used this method [51,52,66], future studies might consider including multiple measurements of pubertal timing, for example by explicitly asking the child and a parent about the child’s pubertal timing [8].

Third, although not a limitation *per se*, it should be acknowledged that the present sample was not drawn from a clinical population with high levels of anxiety and depression. As such, although one can be confident about inferences concerning a potential effect of alexithymia on depression and anxiety in a typical sample with anxiety and depression scores largely in the normal range, one must be cautious about making inferences about clinical samples. It should be noted, however, that alexithymia has been linked to symptoms of depression and anxiety in clinical groups, and that the RCADS is considered a clinical screening tool. It is therefore perfectly possible that alexithymia has the potential to explain the relationship between developmental factors and clinical levels of anxiety and depression as it does for sub-clinical levels of anxiety and depression.

Fourth, future studies are necessary to confirm the potential role of alexithymia in the development of symptoms of depression and anxiety. In the current study we used several measures of specific forms of anxiety, resulting in detailed information concerning specific symptoms, but which also resulted in a relatively large number of analyses. These future studies should use a longitudinal design in order to provide causal evidence for the mechanism proposed here.

### Potential mechanism

The finding that alexithymia may explain some of the developmental influences on symptoms of depression and anxiety during adolescence is consistent with a previous suggestion that alexithymia is a marker for interoceptive impairment, and that interoceptive impairment is responsible for a range of clinical symptoms [59]. Interoception refers to the perception of the internal state of one’s body [67,68], and is thought to underpin a range of emotional processes, including emotional memory [69], emotional stability [70], emotion regulation [71], and emotional intensity [71,72]. Accurate perception of interoceptive information is also thought to be crucial for learning and decision-making, as these processes rely on perception of internal signals of punishment and reward (e.g. [73]). Given the reliance on interoception of fundamental processes such as emotion processing and learning and memory, it is perhaps unsurprising that a number of clinical conditions have been found to be, or theorised to be, characterised by atypical interoception [74–76], including depression and anxiety [77]. Importantly, alexithymia has also been shown to be associated with impaired interoception [78,79], making it possible that developmental factors (e.g., physical change) during adolescence give rise to atypical interoception (signalled by alexithymic traits), which in turn gives rise to clinical symptoms of depression and anxiety. Furthermore, the observation that alexithymic traits contributed towards clinical symptoms in females only is also consistent with previous evidence that females often present with poor objective interoceptive accuracy in comparison to men [79,80], and suggests that the negative impact of atypical interoception on mental health may be more pronounced in females. While this hypothesis is certainly speculative, it is worthy of future investigation using a full battery of tests of interoception during adolescence [78].

### Conclusions

In this study, we aimed to investigate the development of the separate subscales of alexithymia across adolescence, and to explore the potential role of alexithymia in the development of symptoms of depression and anxiety, separately for males and females. Results demonstrated that females with more mature pubertal status experience more difficulties in identifying and describing feelings. This relationship was not found in males. In females, difficulty identifying feelings could explain the effect of pubertal factors on symptoms of depression and anxiety. The current findings suggest that it is important to consider changes in alexithymia when studying the onset and development of internalizing psychological disorders during adolescence.

## Supporting information

S1 Table Overview of participants’ minimum, maximum, and mean scores on measures of depression and anxiety, including the lowest and highest possible scores. (DOCX)

S2 Table The results of the hierarchical regression predicting psychiatric symptoms from maturation measures (age, pubertal stage, pubertal timing) and alexithymia in females and males. (DOCX)

S3 Table The results of the robust regressions predicting psychiatric symptoms from maturation measures (age, pubertal stage and pubertal timing) and factors of alexithymia in females. (DOCX)

S4 Table The results of the robust regressions predicting psychiatric symptoms from maturation measures (pubertal stage and pubertal timing) and factors of alexithymia in males. (DOCX)

S1 Fig Distribution of residuals in all conducted hierarchical regressions. As these distributions are not perfect, especially within the analyses performed in males, we performed robust regression analyses to confirm our results.
(DOCX)

S1 File Supplemental methods and results. (DOCX)
