# Supplementary material for: Alexithymic traits can explain the association between puberty and symptoms of depression and anxiety in adolescent females
Source: PLoS One. 2019 Jan 16;14(1):e0210519. doi: 10.1371/journal.pone.0210519 (PMC6334924; doi:10.1371/journal.pone.0210519)
Supplement: S1 Table — (DOCX) [file pone.0210519.s001.docx]

**S1 Table.**

Overview of participants’ minimum, maximum, and mean scores on measures of depression and anxiety, including the lowest and highest possible scores.

|  | Lowest-Highest possible score | **Females** | | | | **Males** | | | |
| --- | --- | --- | --- | --- | --- | --- | --- | --- | --- |
|  |  | **Min** | **Max** | **Mean** | **SD** | **Min** | **Max** | **Mean** | **SD** |
| **MD** | 0-30 | 0 | 18 | 5.88 | 3.71 | 0 | 16 | 5.22 | 3.86 |
| **GA** | 0-18 | 0 | 12 | 5.23 | 2.64 | 0 | 9 | 3.83 | 2.14 |
| **SP** | 0-27 | 0 | 23 | 8.87 | 4.90 | 0 | 21 | 5.89 | 3.61 |
| **PD** | 0-27 | 0 | 12 | 3.30 | 3.03 | 0 | 8 | 1.75 | 1.82 |
| **SA** | 0-21 | 0 | 12 | 2.69 | 2.23 | 0 | 7 | 1.37 | 1.51 |
| **OCD** | 0-18 | 0 | 10 | 2.08 | 2.39 | 0 | 7 | 2.05 | 1.96 |

*Note.* MD=Major Depression, GA=Generalized Anxiety, SP=Social Phobia, PD=Panic Disorder, SA=Separation Anxiety, OCD=Obsessive Compulsive Disorder
