# Supplementary material for: Alexithymic traits can explain the association between puberty and symptoms of depression and anxiety in adolescent females
Source: PLoS One. 2019 Jan 16;14(1):e0210519. doi: 10.1371/journal.pone.0210519 (PMC6334924; doi:10.1371/journal.pone.0210519)
Supplement: S2 Table — (DOCX) [file pone.0210519.s002.docx]

**S2 Table.**

The results of the hierarchical regression predicting psychiatric symptoms from maturation measures (age, pubertal stage, pubertal timing) and alexithymia in females and males

| **FEMALES** | | | | | | | | | |
| --- | --- | --- | --- | --- | --- | --- | --- | --- | --- |
| Generalized Anxiety | | Beta | *T* | *p* | *R* | *R²* | *R²*change | *F*change | Sig *F*change |
| Model 1 |  |  |  |  | .268 | .072 |  | *5.789* | .019 |
|  | Age | .268 | 2.41 | .019 |  |  |  |  |  |
| Model 2 |  |  |  |  | .598 | .358 | .286 | 10.703 | .000 |
|  | Age | .179 | 1.68 | .098 |  |  |  |  |  |
|  | **DIF** | **.549** | **4.94** | **.000** |  |  |  |  |  |
|  | DDF | -.054 | -.48 | .632 |  |  |  |  |  |
|  | EOT | -.117 | -1.09 | .278 |  |  |  |  |  |
| Major Depression | | Beta | *T* | *p* | *R* | *R²* | *R²*change | *F*change | Sig *F*change |
| Model 1 |  |  |  |  | .330 | .109 |  | 9.191 | .003 |
|  | PDS | .330 | 3.032 | .003 |  |  |  |  |  |
| Model 2 |  |  |  |  | .579 | .336 | .226 | 8.182 | .000 |
|  | **PDS** | **.213** | **2.011** | **.048** |  |  |  |  |  |
|  | **DIF** | **.539** | **4.735** | **.000** |  |  |  |  |  |
|  | DDF | -.132 | -1.142 | .257 |  |  |  |  |  |
|  | EOT | -.067 | -.669 | .506 |  |  |  |  |  |
| Generalized Anxiety | | Beta | *T* | *p* | *R* | *R²* | *R²*change | *F*change | Sig *F*change |
| Model 1 |  |  |  |  | .319 | .102 |  | 8.474 | .005 |
|  | PDS | .319 | 2.911 | .005 |  |  |  |  |  |
| Model 2 |  |  |  |  | .595 | .354 | .252 | 9.379 | .000 |
|  | PDS | .160 | 1.531 | .130 |  |  |  |  |  |
|  | **DIF** | **.532** | **4.739** | **.000** |  |  |  |  |  |
|  | DDF | -.079 | -.691 | .492 |  |  |  |  |  |
|  | EOT | -.156 | -1.566 | .122 |  |  |  |  |  |
| Social Phobia | | Beta | *T* | *p* | *R* | *R²* | *R²*change | *F*change | Sig *F*change |
| Model 1 |  |  |  |  | .229 | .053 |  | 4.159 | .045 |
|  | PDS | .229 | 2.039 | .045 |  |  |  |  |  |
| Model 2 |  |  |  |  | .521 | .272 | .219 | 7.228 | .000 |
|  | PDS | .056 | .502 | .617 |  |  |  |  |  |
|  | **DIF** | **.414** | **3.475** | **.001** |  |  |  |  |  |
|  | DDF | .102 | .839 | .404 |  |  |  |  |  |
|  | EOT | -.117 | -1.106 | .272 |  |  |  |  |  |
| Major Depression | | Beta | *T* | *p* | *R* | *R²* | *R²*change | *F*change | Sig *F*change |
| Model 1 |  |  |  |  | .299 | .089 |  | 7.363 | .008 |
|  | P.Timing | .299 | 2.714 | .008 |  |  |  |  |  |
| Model 2 |  |  |  |  | .576 | .332 | .242 | 8.692 | .000 |
|  | P.Timing | .195 | 1.890 | .063 |  |  |  |  |  |
|  | **DIF** | **.535** | **4.671** | **.000** |  |  |  |  |  |
|  | DDF | -.134 | -1.149 | .254 |  |  |  |  |  |
|  | EOT | -.137 | -1.404 | .165 |  |  |  |  |  |

**MALES**

| Separation  Anxiety | | Beta | *T* | *p* | *R* | *R²* | *R²*change | *F*change | Sig *F*change |
| --- | --- | --- | --- | --- | --- | --- | --- | --- | --- |
| Model 1 |  |  |  |  | .420 | .176 |  | 13.073 | .001 |
|  | PDS | -.420 | -3.616 | .001 |  |  |  |  |  |
| Model 2 |  |  |  |  | .492 | .242 | .066 | 1.680 | .181 |
|  | **PDS** | **-.475** | **-3.420** | **.001** |  |  |  |  |  |
|  | DIF | .144 | 1.075 | .287 |  |  |  |  |  |
|  | DDF | .154 | 1.117 | .269 |  |  |  |  |  |
|  | EOT | .023 | .171 | .865 |  |  |  |  |  |
| Separation Anxiety | | Beta | *T* | *p* | *R* | *R²* | *R²*change | *F*change | Sig *F*change |
| Model 1 |  |  |  |  | .363 | .131 |  | 9.231 | .003 |
|  | P.Timing | -.363 | -3.038 | .003 |  |  |  |  |  |
| Model 2 |  |  |  |  | .449 | .202 | .071 | 1.710 | .175 |
|  | **P.Timing** | **-.363** | **-2.860** | **.006** |  |  |  |  |  |
|  | DIF | .131 | .960 | .341 |  |  |  |  |  |
|  | DDF | .096 | .690 | .493 |  |  |  |  |  |
|  | EOT | .152 | 1.206 | .233 |  |  |  |  |  |

*Note.* In females, the alexithymia factor difficulties identifying feelings was the only predictor of psychiatric symptoms, with maturation measures not a significant predictor after the inclusion of alexithymia factors. In males, maturation measures remained significant predictors even after the inclusion of alexithymia factors. PDS = Pubertal stage: scores from the pubertal development scale. Pubertal timing = scores from the pubertal development scale relative to peers of the same age. DDF = difficulties describing feelings. DIF = Difficulties identifying feels. EOT = externally orientated thinking. Significant predictors are highlighted bold.
