# Supplementary material for: Alexithymic traits can explain the association between puberty and symptoms of depression and anxiety in adolescent females
Source: PLoS One. 2019 Jan 16;14(1):e0210519. doi: 10.1371/journal.pone.0210519 (PMC6334924; doi:10.1371/journal.pone.0210519)
Supplement: S3 Table — (DOCX) [file pone.0210519.s003.docx]

**S3 Table.**

The results of the robust regressions predicting psychiatric symptoms from maturation measures (age, pubertal stage and pubertal timing) and factors of alexithymia in females

| Outcome variable: Major Depression | | | |
| --- | --- | --- | --- |
|  | *b* | *T* | *p* |
| Constant | -1.75 | -0.562 | 0.576 |
| Pubertal stage | 0.22 | 1.836 | 0.070 |
| DDF | -0.19 | -1.046 | 0.299 |
| **DIF** | **0.64** | **4.245** | **<0.001** |
| EOT | -0.11 | -0.785 | 0.435 |
| Outcome variable: Generalized Anxiety | | | |
|  | *b* | *T* | *p* |
| Constant | 1.161 | 0.530 | 0.598 |
| Pubertal stage | 0.120 | 1.455 | 0.150 |
| DDF | -0.066 | -0.514 | 0.609 |
| **DIF** | **0.436** | **4.140** | **<0.001** |
| EOT | -0.169 | -1.752 | 0.084 |
| Outcome variable: Social Phobia | | | |
|  | *b* | *T* | *p* |
| Constant | 5.241 | 1.348 | 0.182 |
| Pubertal stage | 0.095 | 0.649 | 0.519 |
| DDF | 0.124 | 0.540 | 0.591 |
| **DIF** | **0.596** | **3.189** | **0.002** |
| **EOT** | **-0.475** | **-2.774** | **0.007** |
| Outcome variable: Major Depression | | | |
|  | *b* | *T* | *p* |
| Constant | 2.822 | 1.049 | 0.298 |
| Pubertal timing | 0.257 | 1.642 | 0.105 |
| DDF | -0.190 | -1.028 | 0.308 |
| **DIF** | **0.629** | **4.175** | **<0.001** |
| EOT | -0.197 | -1.488 | 0.141 |
| Outcome variable: Generalized Anxiety | | | |
|  | *b* | *T* | *p* |
| Constant | -0.406 | -0.145 | 0.885 |
| Age | 0.152 | 1.526 | 0.131 |
| DDF | -0.043 | -0.341 | 0.734 |
| **DIF** | **0.454** | **4.338** | **<0.001** |
| EOT | -0.128 | -1.231 | 0.222 |

*Note.* The alexithymia factor difficulties identifying feelings was the only predictor of psychiatric symptoms, with maturation measures not a significant predictor after the inclusion of alexithymia factors. Pubertal stage = scores from the pubertal development scale. Pubertal timing = scores from the pubertal development scale relative to peers of the same age. DDF = difficulties describing feelings. DIF = Difficulties identifying feels. EOT = externally orientated thinking. Significant predictors are highlighted bold.
