# Supplementary material for: Alexithymic traits can explain the association between puberty and symptoms of depression and anxiety in adolescent females
Source: PLoS One. 2019 Jan 16;14(1):e0210519. doi: 10.1371/journal.pone.0210519 (PMC6334924; doi:10.1371/journal.pone.0210519)
Supplement: S4 Table — (DOCX) [file pone.0210519.s004.docx]

**S4 Table.**

The results of the robust regressions predicting psychiatric symptoms from maturation measures (pubertal stage and pubertal timing) and factors of alexithymia in males

| Outcome variable: Separation anxiety | | | |
| --- | --- | --- | --- |
| Predictors | *b* | *t* | *p* |
| Constant | 2.025 | 1.430 | 0.158 |
| **Pubertal stage** | **-0.146** | **-3.015** | **0.004** |
| DDF | 0.098 | 1.337 | 0.186 |
| DIF | 0.045 | 0.639 | 0.525 |
| EOT | 0.001 | 0.016 | 0.987 |
| Outcome variable: Separation anxiety | | | |
| Predictors | *b* | *t* | *p* |
| Constant | -0.675 | -0.645 | 0.5213 |
| **Pubertal timing** | **-0.163** | **-2.570** | **0.0128** |
| DDF | 0.075 | 1.046 | 0.2999 |
| DIF | 0.047 | 0.673 | 0.5038 |
| EOT | 0.053 | 0.814 | 0.4188 |

*Note.* Maturation measures remained significant predictors even after the inclusion of alexithymia factors. Pubertal stage = scores from the pubertal development scale. Pubertal timing = scores from the pubertal development scale relative to peers of the same age. DDF = difficulties describing feelings. DIF = Difficulties identifying feels. EOT = externally orientated thinking. Significant predictors are highlighted bold.
