# Supplementary material for: Alexithymic traits can explain the association between puberty and symptoms of depression and anxiety in adolescent females
Source: PLoS One. 2019 Jan 16;14(1):e0210519. doi: 10.1371/journal.pone.0210519 (PMC6334924; doi:10.1371/journal.pone.0210519)
Supplement: S1 Fig — As these distributions are not perfect, especially within the analyses performed in males, we performed robust regression analyses to confirm our results. (DOCX) [file pone.0210519.s005.docx]

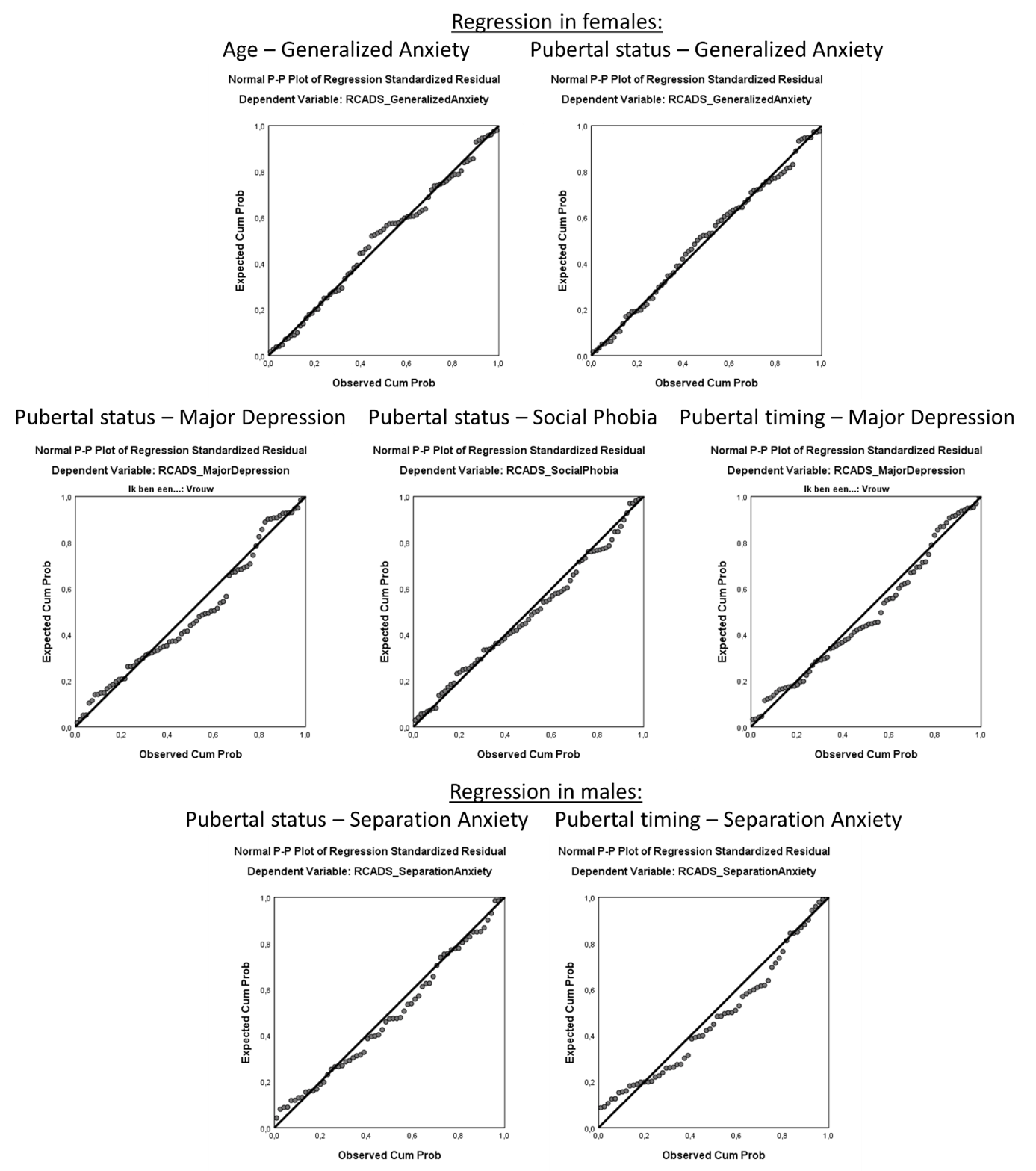
 **S1 Figure.** Distribution of residuals in all conducted hierarchical regressions. As these distributions are not perfect, especially within the analyses performed in males, we performed robust regression analyses to confirm our results.
