# Supplementary material for: Alexithymic traits can explain the association between puberty and symptoms of depression and anxiety in adolescent females
Source: PLoS One. 2019 Jan 16;14(1):e0210519. doi: 10.1371/journal.pone.0210519 (PMC6334924; doi:10.1371/journal.pone.0210519)
Supplement: S1 File — (DOCX) [file pone.0210519.s006.docx]

**S1 File**

**Supplemental methods and results**

**Supplemental methods**

Multiple mediation analyses were conducted in SPSS 23 using the computational tool PROCESS (Hayes, 2013) for females and males separately. For indirect effects, 95% (two-tailed) bias-corrected bootstrapped confidence intervals were calculated using 10,000 repetitions (Streukens and Leroi-Werelds, 2016). An indirect effect is significant if the confidence intervals for the indirect effect do not include zero (Preacher and Hayes, 2004). For the purpose of clarity, we first report the results of all mediation analyses with age, then with pubertal status, and then pubertal timing. Unstandardized coefficients are reported for all mediation analyses.

**Supplemental results**

Mediations analyses were used to test whether alexithymic traits can explain the relationship between developmental factors (age/pubertal status/pubertal timing) and symptoms of depression and anxiety. Analyses were only performed where there were significant correlations between alexithymia and developmental factors, alexithymia and symptom levels, and developmental factors and symptom levels. Note that separate mediation analyses were performed for each developmental variable (age, pubertal status, pubertal timing), and for each of the different RCADS subscales, where the pattern of zero-order correlations made a mediation possible.

**Females.** In females, we tested for mediating effects of alexithymia on the relationship between pubertal status and major depression, generalized anxiety symptoms, and social phobia scores. Next, we tested for mediating effects of alexithymia on the relationship between pubertal timing and depression. Subsequently, we tested for possible mediating effects of alexithymia on the relationship between age and generalized anxiety symptoms.

We found four mediating effects, all including DIF. First, more mature pubertal status was associated with greater difficulties identifying feelings, which in turn was associated with higher rates of depressive symptoms (*Indirect effect of DIF*=.146, *SE*=.07, CI=[.033, .328]). The model indicated a partial mediation, meaning that the relationship between pubertal status and depressive symptoms was still significant when alexithymia was included in the model (*Direct effect* =.224, *SE*=.11, *p*=.048).

Second, a full mediation of the effect of pubertal status on generalized anxiety symptoms by DIF (*Indirect effect of DIF*=.102, *SE*=.05, CI=[.026, .219]) showed that increasing pubertal status was related to more difficulties identifying feelings, which was associated with more generalized anxiety (*Direct effect* =.119, *SE*=.08, *p*=.130) (Figure 1b).

Third, more mature pubertal status was associated with greater difficulties identifying feelings, which in turn was associated with higher rates of social phobia symptoms. This again was a full mediation (*Indirect effect of DIF*=.148, *SE*=.08, CI=[.029, .344]; *Direct effect* =.078, *SE*=.15, *p*=.617).

Fourth, we found a full mediation between pubertal timing and symptoms of depression, showing that earlier pubertal timing was associated with greater difficulties identifying feelings, which was in turn associated with more depressive symptoms (*Indirect effect of DIF*=.218, *SE*=.11, CI=[.032, .485]; *Direct effect* =.281, *SE*=.15, *p*=.063).

Fifth, there was no mediation in the model predicting generalized anxiety from age (*Indirect effect of EOT*=.047, *SE*=.05, CI=[-.047, .166]; *Direct effect* =.157, *SE*=.09, *p*=.098).

**Males.** In males we tested for possible mediating effects of alexithymia on the effect of 1) pubertal status on separation anxiety, and 2) pubertal timing on separation anxiety. Results showed that neither the effect of pubertal status on separation anxiety (*Direct effect* =-.169, *SE*=.05, *p*=.00; all indirect effects were non-significant, i.e. confidence intervals included zero) nor the effect of pubertal timing on separation anxiety (*Direct effect* =-.190, *SE*=.07, *p*=.01; all indirect effects were non-significant) were mediated by alexithymia.
